# HPV16 AND EXPRESSION OF PROTEIN P16^INK4A^ AND E7 ONCOPROTEIN IN COLORECTAL CARCINOMA

**DOI:** 10.1590/0102-672020210002e1637

**Published:** 2022-01-31

**Authors:** Olavo Magalhães PICANÇO-JUNIOR, Thérèse Rachell THEODORO, Paulo José de Brito Silva ALBUQUERQUE, Rodrigo Nascimento PINHEIRO, Jaques WAISBERG

**Affiliations:** 1 Universidade Federal de São Paulo, Programa de pós graduação em Ciência Cirúrgica interdisciplinar - São Paulo - São Paulo - Brasil; 2 Universidade Federal do Amapá - Macapá - AP - Brasil; 3 Faculdade de Medicina do ABC, Centro de Biologia Molecular - Santo André - São Paulo - Brasil

**Keywords:** Colorectal Neoplasms, Papillomavirus Infections, Genes, p16, Papillomavirus E7 Proteins, Neoplasias Colorretais, Infecções por Papillomavirus, Genes p16, Proteínas E7 de Papillomavirus

## Abstract

Human papillomavirus (HPV) is the agent of the most prevalent sexually transmitted diseases in the world associated with cervix and anal canal cancer. The action of HPV on colorectal carcinogenesis is not yet established.

**OBJECTIVE:**

This research aimed to study the possible correlation between the presence of HPV16 and the gene expression of p16^INK4a^ protein and HPV E7 oncoprotein and their levels in colorectal carcinoma tissue.

**METHODS:**

A retrospective case-control study of 79 patients with colorectal carcinoma was divided into two groups: HPV-positive and HPV-negative. The polymerase chain reaction was performed, in addition to dot-blot hybridization for HPV16 and HPV18. Colorectal tissue samples were also subjected to immunohistochemical study to assess the tissue level of E7 and p16^INK4a^ proteins.

**RESULTS:**

HPV was identified in 36 (45.6%) cases. There was no significant difference between groups regarding gender (p=0.056), age (p=0.1), colic and/or rectal location (0.098), and presence of HPV. Gene expression of HPV E7 oncoprotein was present in 3.12% of cases (p=0.9), and p16^INK4a^ protein expression was observed in 46.3% (p=0.27) of those selected with HPV detection.

**CONCLUSION:**

Gene expression and tissue levels of E7 oncoprotein and p16^INK4a^ protein found in HPV-positive patients suggest the absence of HPV16 oncogenic activity in colorectal carcinoma.

## RESUMO - INTRODUÇÃO

O papilomavírus humano (HPV) é agente das doenças sexualmente transmissíveis de maior prevalência no mundo que estão associadas ao câncer do colo do útero e canal anal. A ação do HPV na carcinogênese colorretal não está ainda estabelecida.

**OBJETIVO:**

Estudar a eventual correlação entre a presença do HPV tipo 16 e a expressão gênica da proteína p16^INK4a^ e da oncoproteína E7 de HPV e de seus níveis no tecido do carcinoma colorretal.

**METODOS:**

Estudo retrospectivo caso-controle de 79 doentes com carcinoma colorretal divididos em dois grupos: HPV presente e HPV ausente. Foi realizada reação em cadeia da polimerase (PCR), além da hibridização do tipo *dot blot* para o HPV 16 e o HPV 18 Amostras do tecido colorretal também foram submetidas ao estudo imuno-histoquimico para avaliar o nível tecidual das proteínas E7 e p16^INK4a^.

**RESULTADOS:**

O HPV foi identificado em 36 (45,6%) casos. Não houve diferença significante entre os grupos quanto ao sexo (p=0,056), idade (p=0,1), localização cólica e/ou retal (0,098) e presença do HPV. A expressão gênica da oncoproteína E7 de HPV estava presente em 3,12% dos casos (p=0,9) e a expressão da proteína p16^INK4a^ foi observada em 46,3% (p=0,27) dos indivíduos com detecção do HPV.

**CONCLUSÃO:**

A expressão gênica e os níveis teciduais da oncoproteína E7 e da proteína p16^INK4a^ encontrados nos pacientes positivos para o HPV sugerem a ausência de atividade oncogênica do HPV tipo 16 no carcinoma colorretal.

## INTRODUCTION

Colorectal cancer (CRC) is one of the most frequent neoplasms worldwide and is the third most incident cancer (10.2%) after malignant lung and breast neoplasms^5^ ^,^ ^30^. The CRC is the second one with the highest mortality worldwide and was responsible for 881,000 deaths in 2018 with about 1.8 million new cases^5^ ^,^ ^30^.

Infectious diseases were responsible for 2.2 million new cases of cancer in the world in 2018, representing 13% of all cancer cases, excluding nonmelanoma skin neoplasms^18^. Among the malignant neoplasms with infectious etiopathogenesis, *Helicobacter pylori* was the infectious agent responsible for 810,000 new cases of gastric cancer, and human papillomavirus (HPV) was responsible for 690,000 new cases of cancer affecting mainly the cervix^18^.

HPV is one of the most prevalent sexually transmitted diseases worldwide, and HPV infection is associated with cervix cancer^9^ ^,^ ^20^. Peder et al.^20^ performed a meta-analysis on the association between HPV and noncervical genital cancer with a combined sample of 1,552 patients. These authors verified the presence of HPV in 42% cases of penile cancer, 67% cases of CRC, and 43% cases of vulvar cancer. Other studies^1^ ^,^ ^4^ ^,^ ^7^ ^,^ ^9^ ^,^ ^19^ ^,^ ^22^ correlated HPV with the development of colic adenocarcinoma and, more often, with anal canal cancer. In patients with anal canal tumors, a high proportion of hyperexpression of the *p16* ^*INK4a*^ gene was identified, suggesting that HPV infection is one of the determinants for the development of anal cancer in men and women^1^ ^,^ ^4^ ^,^ ^7^ ^,^ ^9^ ^,^ ^19^ ^,^ ^22^. Some authors have shown the presence of HPV in tumors localized in different locations^6^ ^,^ ^8^ ^,^ ^11^ ^,^ ^23^ ^,^ ^25^ ^,^ ^29^.

The expression of HPV E6 and E7 oncoproteins is related to the development of cervical, oral, and anal canal carcinomas^1^ ^,^ ^23^ ^,^ ^25^. The results presented in a staining protocol for the immunohistochemical evaluation of the expression of oncoproteins E6 and E7 in the cervix and oropharyngeal cancer positive for HPV showed the advantages of this method in comparison with the mRNA hybridization of the ER and E7 proteins due to lower cost and greater applicability in clinical practice^1^ ^,^ ^8^ ^,^ ^23^ ^,^ ^25^.

 In CRC, a substitute marker for the presence of *HPV E7* gene expression was sought by immunohistochemical examination of p16^INK4a^ oncoprotein^13^ ^,^ ^14^. However, the p16^INK4a^ oncoprotein did not indicate a substitute marker for an active HPV infection in CRC, unlike what it is observed in squamous cell cancer of the head and neck tumors and cervix cancer^11^ ^,^ ^25^.

Damin et al. ^9^ identified HPV-positive in 60% of patients with CRC, and type 16, detected in 68.3% of cases, was the most frequent. This finding indicated that this virus could be related to the pathogenesis of CRC, which was also suggested by other authors^4^ ^,^ ^7^ ^,^ ^22^.

However, it is still necessary to define whether the presence of HPV found in tissue samples obtained in patients with CRC determines expressive levels of the E7 oncoprotein and of the HPV p16^INK4a^ protein, an event that could present itself as an important causal association factor for the development of CRC^11^ ^,^ ^24^.

The identification of HPV-DNA in tissue samples from CRC suggests a possible relation of HPV in colorectal carcinogenesis. However, the effective performance of HPV depends on the action of its oncoproteins. Based on this fact, it was considered necessary to evaluate the activity of the E7 oncoprotein in our samples.

This study aimed to verify the possible correlation between the presence of HPV16 and its association with the expression of the *E7* gene of HPV16 and the levels of the protein p16^INK4a^ and the E7 oncoprotein in the CRC tissue.

## METHODS

We performed all studies involving human participants in accordance with the ethical standards of the Institutional Research Ethics (number: 1377/08 and 1.461.817).

In this retrospective and analytical case-control study, it was analyzed 82 CRC tissue samples obtained during elective surgeries with curative-intent treatment for CRC. A polymerase chain reaction (PCR) was performed with human β-globin gene primers to assess the sufficiency and integrity of the DNA present in each sample, with overall three samples being used^3^.

The PCR was performed with generic and specific primers for papillomaviruses 16 and 18 and hybridization in dots (dot blot).

The samples consisted of paraffin-embedded blocks of colorectal tissue obtained from patients with CRC, of both genders, who aged 28-87 years (mean 57.85±15.3 years) and underwent the surgical procedure at the Hospital Ophir Loyola (Belém, Brazil), from January 1999 to December 2003. All patients underwent surgery consecutively during this period and were classified according to the TNM system (American Joint Committee on Cancer).

Colorectal biopsy and imaging exams (chest X-ray, abdominal and pelvic CT scan, and/or abdominal ultrasound) were used to define the diagnosis and staging according to the service protocol, the intraoperative findings, and the anatomopathological examination report of the surgical specimen.

Inclusion criteria were as follows: adult patients, of both sexes, with CRC confirmed by histopathological examination, and who underwent surgery at Hospital Ophir Loyola.

 Exclusion criteria were as follows: patients with hereditary colorectal polyposis syndrome, hereditary nonpolypoid colorectal carcinoma, colorectal inflammatory disease, metachronous CRC, patients undergoing neoadjuvant radiotherapy treatment, and another histological type of CRC other than CRC.

### DNA extraction

Paraffin-embedded tissue sections deposited in a tube were subjected to the process of dewaxing and enzymatic digestion with proteinase K (200 μg/ml) at 56°C for 2-4 days. After digestion, DNA was extracted by the phenol-chloroform method.

### PCR

After extraction and purification, the DNA samples were initially subjected to PCR with the PCO3 and G74 primers that amplify 100 base pairs (bp) of the human β-globin gene to assess the sufficiency and integrity of the DNA present in each sample.

The positive samples were submitted to PCR with generic HPV primers, GP5+/GP6+, capable of amplifying 140 bp of the *HPV L1* gene.

To check the absence of contamination by exogenous DNA, a negative control was used, containing all reagents in the mixture, except DNA. A *HeLa* cell line with integrated HPV18 DNA was used as a positive control.

The amplifications were performed in the thermocycler (Eppendorf Mastercycler Gradiente Model, Germany), with 40 amplification cycles with 1 min for denaturation at 95°C, 1 min for annealing at 55°C, and 1.5 min for chain elongation at 72°C.

The amplification products (amplicons) were analyzed on 7% polyacrylamide gel and stained with silver.

### HPV Identification by hybridization in dots (Dot Blot)

The process for amplicon fixation to the membrane included heating or UV irradiation. Then, the membrane was covered with specific probes (alone or in cocktails) marked with radioactive phosphorus (P32) for HPV types 6, 11, 16, 18, 31, 33, 34, 35, 39, 40, 42, 43, 44, 45, 51, 52, 54, 56, and 58. Hybridization was revealed after exposing the membranes to the RX film for 18-36 h at 70°C. Hybridization with the probe was recognized as evidence that the investigated nucleotide sequence was present in the studied specimen.

In each membrane, in addition to the positive and negative controls of the PCR products, controls were used for different types of HPV, derived from PCR amplification of plasmids and clinical samples.

Subsequently, the membranes were moistened with 2× SSC solution and placed in a plastic bag with 5 ml of 6× SSC solution, 10× Denhardt’s solution, 0.5% SDS, and 100 μg of denatured salmon sperm, and then incubated at 55°C for 3 h (prehybridization).

The radioactive probes were added to the previous solution and incubated at the same temperature for 12-24 h (hybridization). At the end of this period, the membranes were washed with 3× SSC solution and 0.5% SDS in three stages: the first stage for 10 min at room temperature and the other two stages for 30 min each at 55°C. Finally, the membranes were exposed to an X-ray film (X-OmatK-Kodak, Rochester, NY, USA) for 18-36 h at −70°C, and hybridization was checked after the film was developed by the presence of dark spots on the corresponding location to the samples added to the membrane.

### Specific PCR for HPV 16 and 18 E7

The samples were used in a PCR with specific primers for HPV16 and 18 E7 capable of amplifying 217 bp for HPV16 E7 and 137 bp for HPV18 E7. Regarding HPV16, specific primers were used: 5′ GCC CAT TAA CAG GTC TTC C 3′ and 5′ TTT GCA ACC AGA GAC AAC TGA 3′. Regarding HPV18, specific primers were used: 5′ ATG TCA CGA GCA ATT AAG C 3′ and 5′ TTC TGG CTT CAC ACT TCA AAC A 3′.

The amplifications were performed in the thermocycler equipment (Eppendorf Mastercycler Gradiente Model, Germany) in 40 amplification cycles using 1 min for denaturation at 95°C, 1 min for annealing at 55°C, and 1.5 min for chain elongation at 72°C.

The amplification products (amplicons) were analyzed on a 7% silver-stained polyacrylamide gel and analyzed in a similar way to those for generic RT-PCR.

### Immunohistochemical Technique

The paraffin blocks containing the histological specimens were sectioned 3 μm thick and mounted on silanized slides. They were treated with large streptavidin-avidin-biotin (LSAB)-peroxidase (avidin-biotin-peroxidase complex) using the immunohistochemistry technique. Steaming with citrate solution was employed in antigenic recovery. In blocking endogenous peroxidases and nonspecific sites, 10 V hydrogen peroxide and 2% skimmed milk were, respectively, used. The anti-HPV16 E7 N-21 (SC-1588) and anti-P16-ARC FL-151 (SC-68393) monoclonal antibodies were then incubated for 18 h (Santa Cruz Biotechnology Inc^®^, Santa Cruz, CA, USA ) at a 1:100 dilution in bovine serum albumin (Sigma^®^, St Louis, MI, USA). After this period, the slides were incubated with the secondary antibody LSAB-HRP (System Peroxidase; Dako A/S^®^, Copenhagen, Denmark), developed with chromogen 3-3′-diaminobenzidine (Dako A/S^®^), and counterstaining was performed using Harris hematoxylin (Sigma Diagnostics, St. Louis, MI, USA).

### Image Capture and Treatment

**Figure 1 -:**
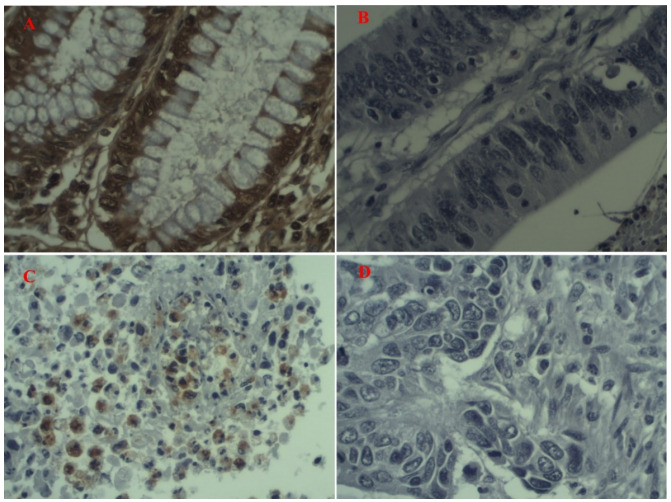
Photomicrographs of immunohistochemistry slides (400×). A - Photomicrograph with p16 ^INK4a^ expression. B - Photomicrograph with no p16 ^INK4a^ expression. C - Photomicrograph with E7 expression. D - Photomicrograph with no E7 expression.

The staining of each marker in the immunohistochemical assay was quantified only in colic and rectal tissues at 100× magnification, using the hotspot principle (identification in the smallest area increased with the highest concentration of histological alteration investigated), while in the 400× increase, the field of greatest expression was captured digitally (Figure 1).

The image capture system used a Nikon Eclipse E200 microscope (Nikon Company, Tokyo, Japan) attached to the digital camera.

The files, saved as tagged image file format (TIFF), were processed using the Corel photo-paint image editing software (Corel Corporation, Ottawa, Canada) using the “color mask” feature initially, where the color pixel predominantly corresponding to the expression of the immunohistochemistry marker was selected.

This edited file was transferred to the Image J image analysis software (imagj.nih.gov/ij/) available on the Windows platform. In this program, the color images were converted into grayscale (color to gray) and then converted again into a binary image, resulting in an image consisting only of white and black pixels, the latter corresponding to the expression of the p16^INK4a^ protein marker and the HPV E7 oncoprotein. The “count black and white pixels” feature quantified in absolute value and, in percentage, the expression of the white and black pixels of each captured histological field.

### Statistical analysis

Chi-square test was used to examine the homogeneity between the proportions, Student’s t-test for independent samples, and Fisher’s exact test in cases where the frequencies were <5. The value of p<0.05 was adopted as the level of significance for the rejection of the null hypothesis. Statistical tests were performed using the BioEstat 5.0 program *(https://www.mamiraua.org.br/documentos/374dcfbeb64a59a98770b581ec51962b.zip).*

## RESULTS

During the research development, three cases were excluded due to negativity for testing the human β-globin gene for HPV DNA identification, which totaled 79 viable cases for the study that showed positivity for the human β-globin gene in 92.9% of samples.

Thus, 79 patients who aged 28-87 years were analyzed, with a mean age of 57.85 ± 15.3 years and a median age of 58 years.

After performing the PCR analysis, patients were divided into two groups, differentiated by the presence or absence of HPV. Thirty-six (45.5%) patients were allocated to the HPV-positive group and 43 (54.5%) patients belonged to the HPV-negative group.

It was observed that there was no significant difference in relation to gender, the age of patients in both groups, and the location of HPV in the cervix or rectum in patients in the group where HPV was present (Table 1).

**Table 1 -:** Characterization of patients operated for colorectal carcinoma in relation to the variables: gender, age, and anatomical location of the collection of the colorectal tissue sample.

Variable	Category	HPV				p
		Positive		Negative		
		n	%	n	%	
Gender	Female	10	27.8	21	48.8	0.056^(1)^
	Male	26	72.2	22	51.2	
Age	<40	3	8.3	4	9.3	1.000^(2)^
	40-49	7	19.4	8	18.6	
	50-59	7	19.4	9	20.9	
	≥60	19	52.8	22	51.2	
Location	RC	6	16.7	14	32.6	0.098
	LC	10	27.8	5	11.6	
	Rectum	20	55.6	24	55.8	

1. Descriptive level of probability of chi-square test.

2. Descriptive level of probability of Fisher’s exact test.

RC: right colon; LC: left colon.

Regarding the TNM stage, clinical staging, and the degree of cell differentiation, no significant difference was observed in relation to the presence or absence of HPV (Table 2).

**Table 2 -:** Characterization of the 79 patients operated for colorectal carcinoma according to the TNM stage, clinical staging, and the degree of cell differentiation, regarding the presence or absence of HPV.

Variable	Category	HPV				p
		Positive		Negative		
		N	%	N	%	
T	1	1	2.85	1	2.32	0.2699
	2	5	14.28	8	18.6	
	3	26	74.28	29	67.44	
	4	3	8.57	5	11.62	
N	0	16	45.57	26	60.49	0.3823
	1	11	31.42	11	25.58	
	2	8	22.85	6	13.95	
M	0	27	77.14	35	81.39	0.1149
	1	8	22.85	8	18.6	
Clinical staging	I	4	11.42	6	13.95	0.3174
	II	9	25.71	18	41.86	
	III	14	40	11	25.58	
	IV	8	22.85	8	18.6	
Differentiation degree	I	4	11.42	8	18.6	0.3420
	II	25	71.42	31	72.09	
	III	6	17.14	4	9.3	

Chi-square test. N = number of patients.

The immunohistochemical study for proteins p16^INK4a^ and E7 did not show a significant difference (p=0.26) in their presence in both groups.

E7 oncoprotein was present only in the group where HPV was detected, and there was no significant difference (p=0.89) in the tissue level of E7 oncoprotein between the group with HPV-positive or HPV-negative (Table 3).

**Table 3 -:** Characterization of patients operated for colorectal carcinoma in relation to the level of HPV p16^INK4a^ and E7 proteins in colorectal tissue.

Variable	HPV		p*
	Positive	Negative	
	%	%	
P16 ^INK4a^	46.311	42.799	0.2699
E7	3.218	0	0.8983

*Student’s t-test.

## DISCUSSION

Studies have been conducted to identify the presence of HPV in the cervix and rectum affected by CRC but still with conflicting results. Pelizzer et al.^21^, when performing a systematic review with meta-analysis, verified the association between HPV and CRC, which was also observed by Damim et al.^10^ In contrast to these data, Vuitton et al.^26^ refuted the relationship of HPV in colorectal carcinogenesis. Picanço Junior et al.^24^ observed the presence of HPV in 46% of patients with CRC, with no relation to the TNM stage, clinical stage, or degree of cell differentiation.

The risk of developing CRC in the population is over 6%^16^. Patients with a family history represent up to 20% of CRC cases, and 5%-10% would be related to the interaction with polypoid and nonpolypoid syndromes. The remaining cases would happen sporadically, representing 80% of CRC^16^.

Thus, the question arises regarding the association between HPV and the development of CRC, since the simple presence of HPV is not considered a factor for the development of cancer and there is a need for the oncogenic activity of the E7 protein of HPV and the inactivity of tumor suppressor genes, such as p16^INK4a^ ^15^ ^,^ ^16^.

This study sought to identify the presence of HPV and to verify the level of viral E7 oncoprotein and the HPV p16 ^INK4a^ protein in the CRC tissue^14^ ^,^ ^27^.

Oncoviruses contribute to carcinogenesis by promoting genetic instability and inducing chromosomal aberrations^27^. In this sense, one can associate the changes induced by the presence of HPV in the development of CRC as a possible causal factor.^27^ However, increasing the level of a particular protein does not necessarily imply an increase in the activity of that protein, which may be functionally inactive^1^ ^,^ ^11^ ^,^ ^25^.

Studies have linked the hyperexpression of the p16^INK4a^ protein with the processes of carcinogenesis and progression of CRC^13^ ^,^ ^28^. Herman et al.^12^ found that reduced expression of the p16^INK4a^ protein is associated with hypermethylation of genes that predispose to CRC in 32%-55% of cases. The loss of the function of the p16^INK4a^ protein resulting from its aberrant methylation was associated with the appearance of several neoplasms, including the CRC, and a close relationship between the positivity of the p16^INK4a^ protein and the manifestation of the CRC was noted^12^ ^,^ ^31^.

Our data indicated that HPV was not associated with CRC, and the level of p16^INK4a^ was not significant as a marker of HPV infection in CRC; similar results similar were observed by Libera et al.^17^

Despite the need for E7 expression for carcinogenesis to occur after HPV integration into the host cell^8^ ^,^ ^11^, its presence alone may not be a definitive factor for progression in tumor development^13^ ^,^ ^16^ ^,^ ^28^. These data are corroborated by the observation that individuals infected with HPV do not necessarily develop tumor cells, which suggest that other sequential events for carcinogenesis are imperative^32^. Thus, the role of HPV in carcinogenesis remains controversial^2^ ^,^ ^9^ ^,^ ^10^ ^,^ ^17^ ^,^ ^24^.

Deschoolmeester et al.^11^ identified the presence of HPV-DNA in 14.2% of tissue samples from patients with CRC, lower than other authors^4^ ^,^ ^9^ ^,^ ^14^ ^,^ ^15^ ^,^ ^24^ who pointed out the presence of HPV in 21%-97% of cases. These discrepancies are attributed to geographic, cultural, and sanitation differences in the regions where the study patients were from.

Regarding the distribution of HPV in the colon, we did not observe any differences regarding the location where the DNA of the virus was detected. These data are consistent with those observed by Damin et al.^9^, which may indicate that contamination by HPV does not occur by retrograde viral transmission from the anogenital region. There were also no significant differences between the clinical and pathological variables studied and the presence or absence of HPV.

In the present investigation, the activity of the tumor suppression protein p16^INK4a^ was not identified, unlike what is observed in cases of HPV-related cervical neoplasia, where this protein acts as a substitute marker for the oncogenic activity of the HPV E7 oncoprotein.

The findings obtained in this study are consistent with data from the current literature in which the presence of HPV is observed in tissue samples from patients with CRC^9^ ^,^ ^11^ ^,^ ^20^ ^,^ ^25^. However, other authors have not evidenced the presence of any type of HPV^16^ ^,^ ^26^, which could be related to the different HPV-DNA identification techniques used in these studies.

Limitations were observed in the course of the present study, such as the number of samples, the difficulty in obtaining adequate material from surgical specimens from paraffin-embedded blocks, and the retrospective nature of the study.

The role played by HPV in the genesis of CRC remains controversial. Further studies are still needed to clarify whether HPV involves, to some degree, in CRC carcinogenesis in patients with this virus detected in CRC tissue.

In conclusion, there were no gene expression and significant levels of HPV E7 oncoprotein and p16^INK4a^ protein in CRC tissues where the presence of HPV16 was detected in this study. These findings suggest the absence of oncogenic activity of HPV16 in CRC.

## CONCLUSION

There was no gene expression and significant levels of HPV E7 oncoprotein and p16^INK4a^ protein in RCC tissues where the presence of HPV type 16 was detected. These findings saved from the oncogenic activity of HPV type 16 in RCC.
